# Achieving High‐Performance Transcranial Ultrasound Transmission Through Mie and Fano Resonance in Flexible Metamaterials

**DOI:** 10.1002/advs.202500170

**Published:** 2025-03-26

**Authors:** Jie Chen, Bing Liu, Genshen Peng, Linming Zhou, Chengwei Tan, Jiale Qin, Juan Li, Zijian Hong, Yongjun Wu, Minghui Lu, Feiyan Cai, Yuhui Huang

**Affiliations:** ^1^ School of Materials Science and Engineering Institute of Fundamental and Transdisciplinary Research State Key Laboratory of Silicon and Advanced Semiconductor Materials Cyrus Tang Center for Sensor Materials and Applications Zhejiang University Hangzhou Zhejiang 310030 China; ^2^ Nanhu Brain‐computer Interface Institute Hangzhou Zhejiang 311100 China; ^3^ College of Electronic Information and Engineering Hangzhou Dianzi University Hangzhou Zhejiang 310018 China; ^4^ Department of Ultrasound Women's Hospital Zhejiang University School of Medicine Hangzhou Zhejiang 310006 China; ^5^ Zhejiang University of Technology College of Materials Science and Engineering Hangzhou 310014 China; ^6^ Zhejiang Key Laboratory of Advanced Solid State Energy Storage Technology and Applications Taizhou Institute of Zhejiang University Taizhou Zhejiang 318000 China; ^7^ Hangzhou City University Hangzhou Zhejiang 310015 China; ^8^ National Laboratory of Solid State Microstructures and Department of Materials Science and Engineering Nanjing University Nanjing Jiangsu 210093 China; ^9^ Paul C. Lauterbur Research Center for Biomedical Imaging Shenzhen Institutes of Advanced Technology Chinese Academy of Sciences Shenzhen 518055 China

**Keywords:** fano resonance, flexible metamaterials, mie resonance, transcranial ultrasound

## Abstract

Transcranial ultrasound holds great potential in medical applications. However, the effective transmission of ultrasound through the skull remains challenging due to the acoustic impedance mismatch, as well as the non‐uniform thickness, and the curved surface. To overcome these challenges, this work introduces an innovative Mie‐resonance flexible metamaterial (MRFM), which consists of periodically arranged low‐speed micropillars embedded within a high‐speed flexible substrate. The MRFM generates Mie‐resonance, which couples with the skull to form Fano resonance, thereby enhancing ultrasound transmittance through the skull. Simulation results demonstrate that the proposed resonance solution significantly increases transcranial ultrasound transmittance from 33.7% to 75.2% at 0.309 MHz. For the fabrication of the MRFM, porous nickel foam is used as the Mie micropillars, and agarose hydrogel serves as the flexible substrate. Experimental results demonstrate enhanced ultrasound transmittance from 20.6% to 73.3% at 0.33 MHz with the MRFM, which shows good agreement with the simulation results, further validating the effectiveness of the design. The simplicity, tunability, and flexibility of the MRFM represent a significant breakthrough, addressing the limitations of conventional rigid metamaterials. This work lays a solid theoretical and experimental foundation for advancing the clinical application of transcranial ultrasound stimulation and neuromodulation.

## Graphical abstract

Transcranial ultrasound holds great potential in medical applications. However, effective transcranial ultrasound transmission remains challenging. To address these, this work introduces an innovative Mie‐resonance flexible metamaterial (MRFM), which consists of periodically arranged low‐speed micropillars embedded within a high‐speed flexible substrate. The MRFM generates Mie‐resonance, which couples with the skull to form Fano resonance, thereby enhancing transcranial ultrasound transmittance.

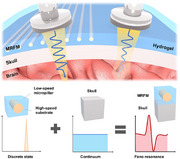

## 1 Introduction

Transcranial ultrasound has significant medical value due to its safety, portability, and potential for long‐term monitoring.^[^ ^1^, ^2^, ^3^, ^4^, ^5^, ^6^, ^7^, ^8^, ^9^ ^]^ It has shown promise in neuromodulation^[^ ^1^, ^2^, ^3^, ^4^, ^5^, ^6^ ^]^ and the treatment of neurological disorders,^[^ ^9^ ^]^ as it typically requires a single‐frequency energy to stimulate targeted areas of neurons in the brain. However, effective ultrasound transmission through the skull remains challenging. The large acoustic impedance mismatch between the skull and intracranial soft tissue leads to significant reflection at the skull‐tissue interface. Furthermore, the non‐uniform thickness, the curved surface, and porous structure of skulls complicate ultrasound propagation, drastically reducing the achievable intensity at target brain regions and compromising therapeutic efficacy.

In recent years, the rapid advancement of acoustic metamaterials has offered novel approaches to the above problems. Acoustic metamaterials are engineered periodic structures composed of subwavelength units that exhibit exceptional physical properties,^[^ ^10^, ^11^, ^12^, ^13^, ^14^, ^15^, ^16^, ^17^, ^18^, ^19^, ^20^, ^21^ ^]^ enabling unique functionalities such as negative refraction,^[^ ^14^, ^15^ ^]^ negative reflection, and acoustic cloaking.^[^ ^16^, ^17^ ^]^ For instance, Shen et al. designed a complementary metamaterial based on transformation acoustics, with simulations indicating its potential for acoustic cloaking for flat skull obstacles, presenting a new solution to enhance the transcranial ultrasound transmittance.^[^ ^18^ ^]^ Li et al. subsequently developed a Helmholtz‐resonance metamaterial, allowing ultrasound waves at 0.113 MHz to maintain the original beam shape after passing through curved, non‐uniform skull models.^[^ ^19^ ^]^ Furthermore, Park et al. designed a Fabry‐Perot (FP) resonance metamaterial that can image complex‐shaped targets located behind 1‐mm‐thick plate steel barriers.^[^ ^20^ ^]^ While these researches have demonstrated the feasibility of acoustic metamaterials in enhancing the transmittance of transcranial ultrasound, several limitations remain. For example, the structural design of metamaterials based on transformation acoustics is highly complex and currently only applicable for low‐frequency ultrasound, while FP resonance‐based metamaterials are limited to plate obstacles, restricting their broader applications.

This work proposes a novel solution to improve the transmittance of transcranial ultrasound based on Fano resonance and Mie resonance. Fano resonance is an asymmetric resonance phenomenon first introduced by Ugo Fano.^[^ ^22^ ^]^ Due to its unique interference effects and high tunability, Fano resonance is widely applied across fields such as quantum science, optics,^[^ ^23^, ^24^, ^25^ ^]^ and acoustics. Mie resonance, on the other hand, was proposed by Gustav Mie while investigating the scattering of light by small spherical particles.^[^ ^26^ ^]^ When the wavelength of the incident wave is close to or smaller than the particle size, different orders of resonance modes are excited within the particle, particularly when it has a relatively high refractive index.^[^ ^27^ ^]^ Mie resonance also finds extensive applications in acoustics,^[^ ^28^, ^29^, ^30^, ^31^, ^32^, ^33^ ^]^ including acoustic absorber,^[^ ^30^, ^31^, ^32^ ^]^ acoustic cloaking and acoustic imaging.

In this work, we propose a Mie‐resonance flexible metamaterial (MRFM) featuring the periodic arrangement of low‐speed micropillars in a flexible high‐speed hydrogel substrate. The MRFM generates Mie‐resonance at a specific frequency *f*_m_, which couples with the skull in a continuous state to form Fano resonance, thereby enhancing ultrasound transmittance through the skull at another specific frequency *f*_0_. Simulation results indicate that the proposed solution increases transcranial ultrasound transmittance from 33.7% to 75.2% at 0.309 MHz for a plate skull model. Furthermore, we select Nickel foam as the Mie micropillars and agarose hydrogel as the flexible matrix to gain the optimal MRFM. Experimental tests demonstrate good consistency with the simulation results. A Fano resonance enhancement peak is observed at 0.33 MHz, and ultrasound scanning tests further confirm the enhanced transcranial transmission effect of the MRFM.

The most significant contribution of this work is its breakthrough in overcoming the limitations of rigid and complexly structured metamaterials. By innovatively coupling MRFM with the skull to form Fano resonance, this work achieves high transcranial ultrasound transmittance at a specific frequency. Notably, MRFM features simple micropillar units, with a working frequency exceeding 0.3 MHz, and the structural parameters (radius, unit length, etc.) can be tailored according to skull properties (density, thickness, etc.). Additionally, MRFM is flexible which allows it fully conform to the curved skull. This advancement lays a robust theoretical and experimental foundation for advancing the clinical application of transcranial ultrasound stimulation and neuromodulation.

## 2 Results and Discussion

### 2.1 High‐Performance Transcranial Ultrasound Transmission Based on Fano Resonance

**Figure 1:**
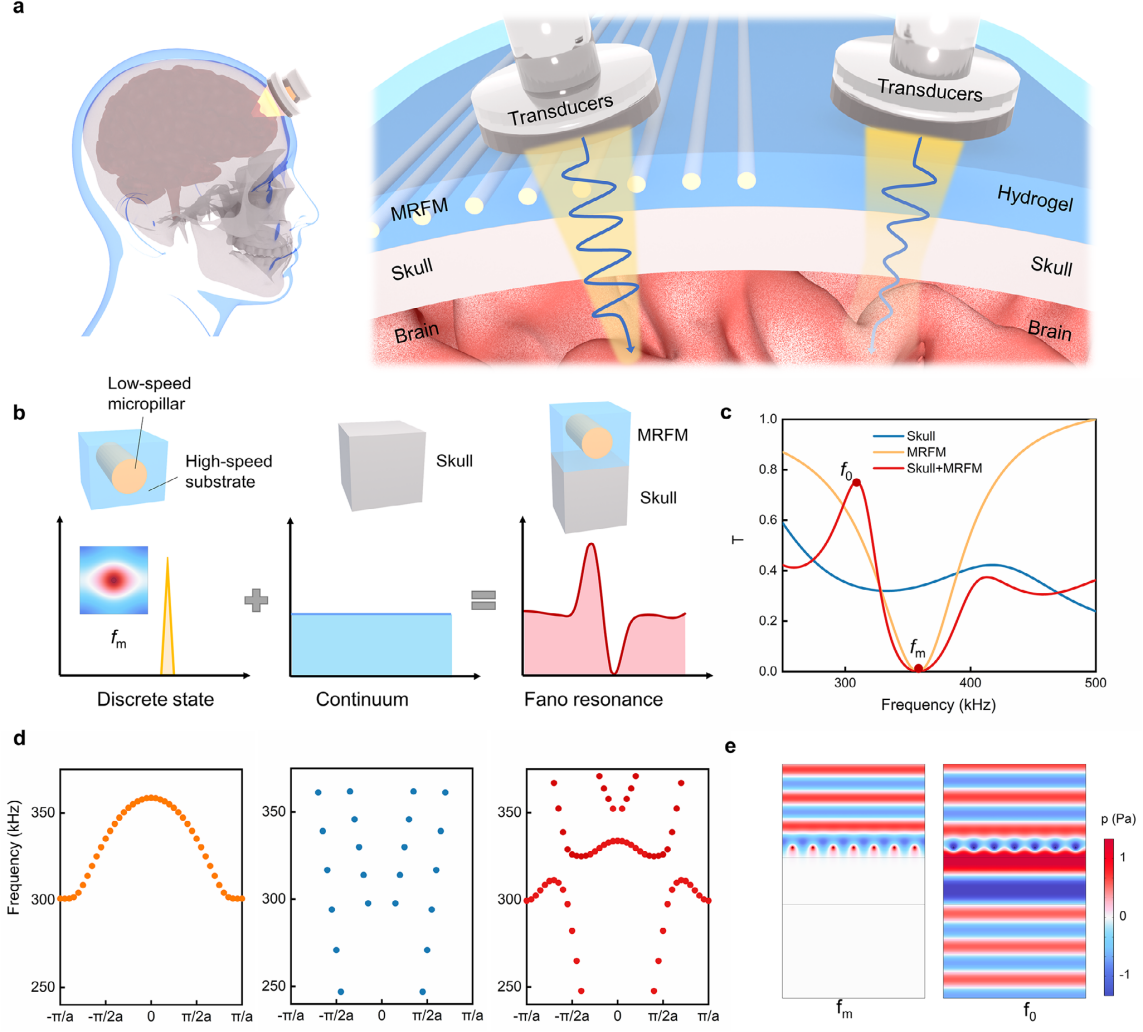
Enhanced transcranial ultrasound transmittance based on Fano resonance. a) Schematic of MRFM's target applications in transcranial ultrasound stimulation. b) The schematic of Fano resonance. c) The ultrasound transmission spectrum in frequency domain. Results for the cases of MRFM only (yellow), skull only (blue) and MRFM installed in front of the skull (red) are plotted. *f*_m_ represents Mie‐resonance frequency, *f*_0_ represents the frequency of maximum transmittance. The acoustic attenuation coefficient is introduced in the skull. See the method for details. d) The band structure of MRFM only (left), skull only (middle), the skull with MRFM (right). e) The sound pressure distribution of ultrasound passing through the skull with MRFM at *f*_m_ and *f*_0_.

Due to the structural and acoustic properties of the skull as described in the introduction, ultrasound encounters significant limitations in achieving high transmittance and precise focusing through the skull, thereby posing challenges for effective intracranial stimulation. **Figure** **1**a illustrates our conceptual vision for how MRFM could be applied to enhance transcranial ultrasound stimulation. The MRFM proposed in this work features a simple structure, customizable design, and flexible adhesion, offering the potential to significantly improve ultrasound transmission through the skull at specific frequencies. This approach underscores the promising role of MRFM in advancing transcranial ultrasound therapy and neuromodulation techniques.

The schematic diagram in Figure 1b illustrates the underlying principles of enhanced ultrasound transmittance achieved by the MRFM. And the transmission spectrum and band structure of (i) MRFM only, (ii) skull only, and (iii) the skull with MRFM are depicted in Figure 1c,d, providing a detailed explanation of the mechanism. The Fano resonance is characterized by a basic scattering resonance with an asymmetric linear pattern, where its unique linearity arises from the interference between the amplitudes of a continuous scattered wave and a discrete excited wave. In this case, the skull facilitates the necessary continuous state of Fano resonance, resulting in a smooth transmission spectrum and a broadband band structure. In contrast, the MRFM accounts for the discrete state necessary for Fano resonance. The MRFM consists of two components, the periodic arrangement of low‐speed micropillars and the flexible high‐speed hydrogel substrate, which generates a variety of resonance modes according to the Mie theory. In our work, the MRFM exhibits distinct monopolar resonance at 358 kHz and dipolar resonance at 546 kHz, as illustrated in Figure S1. The monopolar resonance mode accounts for the discrete state and results in the sharp symmetry peak at *f*_m_ (358 kHz) in the transmission spectrum with a π‐phase shift in the phase spectrum (Figure S2), while the band structure displays narrow band characteristics. The coupling between the discrete excitation wave of the MRFM and the continuous scattering wave of the skull leads to Fano resonance, resulting in an asymmetrical peak in the transmission spectrum of the skull with MRFM. At frequency *f*_m_ (358 kHz), the sound intensity transmittance rapidly drops to zero, and increases sharply to 75% at *f*_0_ (309 kHz), where *f*_0_ denotes the frequency of maximum transmittance. This coupling produces a new energy band gap and generates a novel mode at the edge of the band gap. The sound pressure distribution of ultrasound passing through the skull with MRFM at *f*_0_ and *f*_m_ is illustrated in Figure 1e, showing nearly complete attenuation at *f*_m_ and high transmittance at *f*_0_, thereby further validating the principle of enhancing transmittance for transcranial ultrasound.

### 2.2 Transmission Enhancement Through a Plate Skull Model by MRFM

**Figure 2:**
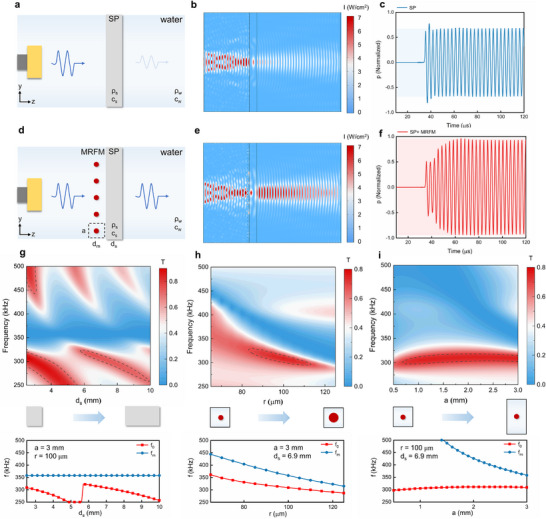
Ultrasound transmission simulations through a plate skull model (SP) with MRFM. a–c) The schematic, the sound intensity distribution and the time‐dependent sound pressure signal of a plane wave at 0.309 MHz through SP only, d–f) through the SP with MRFM. The acoustic attenuation coefficient is ignored in time‐domain simulation. g–i) The contour maps depicting the influence of different skull thickness *d*_s_, micropillar radius *r*, unit length *a* on the ultrasonic transmission spectrum where the MRFM is installed in front of the SP. The attenuation coefficients were retained in this frequency‐domain simulation. The line diagrams of depicting the influence of different skull thickness *d*_s_, micropillar radius *r* and unit length *a* on the *f*_0_ and *f*_m_. (Parameter control protocol: All non‐target parameters are fixed at baseline values from Table S1).

Simulation results in the frequency domain indicate that the MRFM increases transcranial ultrasound transmittance from 33.7% to 75.2% at 0.309 MHz for a plate skull model (SP), as shown in Figure 1c. To further clarify the enhanced ultrasonic transmission characteristics by the MRFM and Fano resonance, we quantified the spatiotemporal evolution of acoustic intensity distributions through the SP with and without the MRFM where the loss effect is ignored. In simulations, the characteristic acoustic impedances of background medium (water or tissue) and skulls are *Z*_w_ = *ρ*_w_ *c*_w_ = 1.50 × 10^6^ Pa·s m^−1^ and *Z*_s_ = *ρ*_s_ *c*_s_ = 3.77×10^6^ Pa·s m^−1^, respectively. Here, the densities for water and the skull are *ρ*_w_ = 1000 kg m^−3^ and *ρ*_s_ = 1370 kg m^−3^, and speeds of sound are *c*_w_ = 1500 m s^−1^ and *c*_s_ = 2751 m s^−1^, respectively. Due to the mismatch in acoustic impedance between the SP and the surrounding medium, the sound intensity attenuates to 45.3% when a plane wave at 0.309 MHz passes through SP only, as shown in the **Figure** **2**a–c. In contrast, the simulation results indicate that when the MRFM is installed in front of SP, the sound intensity remains at 92.0%. And the sound pressure amplitude gradually increases from 49.8% to 95.9% and stabilizes, demonstrating the temporal evolution of transmission enhancement based on Fano resonance. Although the omission of attenuation coefficients leads to elevated overall transmission coefficients in time‐domain analyses compared to frequency‐domain results, it should be emphasized that our primary objective focuses on elucidating the dynamic evolution process. The fundamental physical principles governing wave propagation and the key conclusions regarding the mechanisms of transmission enhancement remain consistent across both analytical dimensions. Additionally, we calculate the sound intensity distribution for the focused ultrasound beam passing through SP with and without the MRFM, which align closely with the plane wave results, as shown in Figure S3.

To further validate the on‐demand design capability of the MRFM, we calculated the transcranial ultrasound transmittance under varying skull thickness *d*_s_, micropillar radius *r* and unit length *a*, as shown in Figure 2g–i. It should be emphasized that when analyzing the impact of a single parameter, all the other parameters are maintained at their default values from Table S1. The area enclosed by the dotted line represents regions where the sound intensity transmittance exceeds 70%. Results show that the frequency *f*_m_ remains unaffected by changes in *d*_s_, whereas *f*_0_ exhibits a periodic trend with increasing *d*_s_. This observation is consistent with the fact that *f*_m_ is determined by the MRFM, and Fano resonance occurs only when the energy of the resonant state matches that of the continuous state. Therefore, it is necessary to tailor the MRFM parameters according to different *d*_s_. However, when *d*_s_ varies within a small range, the same structure of the MRFM can achieve effective transcranial transmission. For instance, at a frequency such as 0.3 MHz, the sound intensity transmittance exceeds 70% for *d*_s_ ranging from 6.5 to 8.1 mm, demonstrating that MRFM effectively enhances ultrasound transmittance through the skull with non‐uniform thickness. Furthermore, Figure 2h provide the contour map depicting the effects of different micropillar radius *r*. The high transmission region (dark red) is close to the low transmission region (dark blue), supporting the asymmetric sharp peak characteristics of Fano resonance. Moreover, both *f*_m_ and *f*_0_ exhibit a linear downward trend as micropillar radius *r* increases. The influence of different unit length *a* is depicted in Figure 2i. While *f*_m_ exhibit a linear downward trend as unit length *a* increase, *f*_0_ stays nearly unchanged. These confirm that within a specific frequency range, *f*_0_ can be tuned by adjusting the MRFM parameters (see other parameters in Figure S4). These results confirm the on‐demand design capabilities of the MRFM. By optimizing the structural parameters of the MRFM based on the SP, it is possible to couple the two to produce Fano resonance and form a transmittance peak. The adjustment of the MRFM enables the determination of the desired high‐transmittance frequency point *f*_0_.

Furthermore, the acoustic properties of the human skull are inherently heterogeneous. We incorporated regions with discrete density (1370 , 1500, and 1800 kg m^−3^) and sound velocity (2751, 2760, and 2770 m s^−1^) profiles, as well as linearly gradual density (from 1370 to 1500 kg m^−3^) and sound velocity (from 2751 to 2800 m s^−1^) distributions, respectively, into a single skull model. Additionally, to account for the three‐layer (compact bone – spongy bone – compact bone) structure of the skull, a significant attenuation coefficient was applied to the intermediate layer in the frequency domain. Under these two distinct modeling conditions, the MRFM demonstrated an enhancement in average acoustic intensity transmittance by 19.8% and 25.3% at *f*_0_, respectively, as depicted in the Figure S5. In addition to characterizing the average acoustic intensity transmittance in the frequency domain, we also conducted a time‐domain analysis to evaluate the transcranial transmission performance where the loss effect is ignored. Nine detection points were strategically designed in different regions behind the heterogeneous skull, enabling a detailed statistical analysis of the transmitted signals, as shown in Figure S6. For the case of SP with discrete density and sound velocity, the average transmittance across nine detection points was 27.5%, with a fluctuation of 7.1%. When MRFM was placed in front of the SP, the average transmittance across nine detection points was 64.7%, with a fluctuation range of 25.1%. Each detection point exhibited at least 19.7% enhancement in transmittance. The discretization of the skull model likely contributed to the observed variations in the enhancement effect across different points. For the case of SP with linearly gradual density and sound velocity, the average transmittance across nine detection points was 31.5%, with a fluctuation range of 8.8%. And the average transmittance across nine detection points reached 75.2% when the MRFM was implemented, with a fluctuation range of 17%. Each detection point demonstrated a minimum transmittance enhancement of 36%. In both models, the MRFM demonstrated a significant enhancement of transmittance behind the skull, despite its inherent heterogeneity in density and sound velocity distribution. It is important to note that incorporating attenuation coefficients remains a challenging task under the constraints of time‐domain simulation conditions. Nevertheless, these findings highlight the robustness and reliability of the MRFM in significantly enhancing ultrasound transmittance under diverse and challenging conditions.

**Figure 3:**
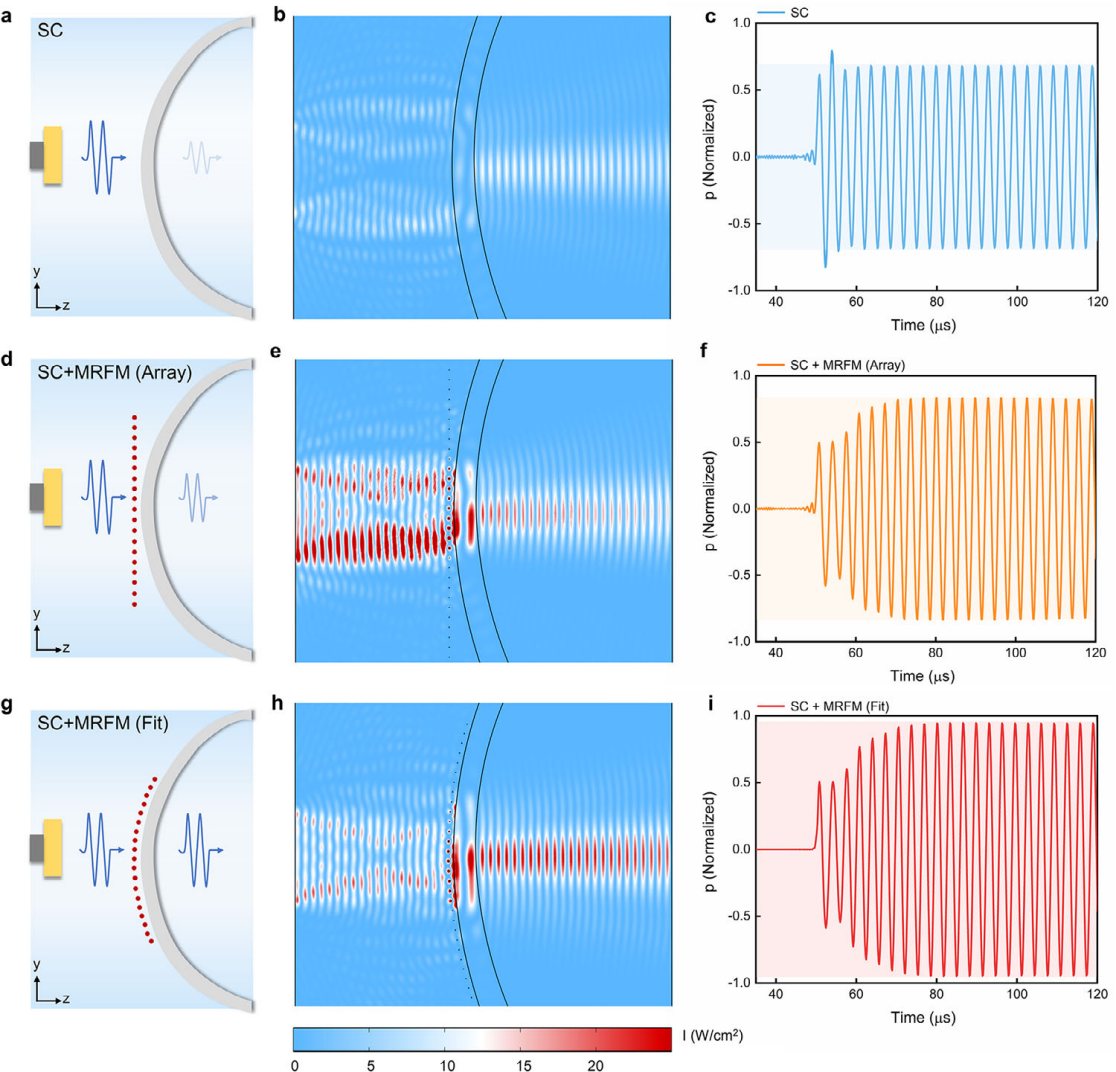
Ultrasound transmission simulations through a curved skull model (SC) with MRFM. a–c) The schematic, the sound intensity distribution, and the time‐dependent sound pressure signal of ultrasonic wave at 0.309 MHz passing through SC only, d–f) through SC with arrayed MRFM. g–i) through SC with fitted MRFM. The acoustic attenuation coefficient is ignored in time‐domain simulation.

### 2.3 Transmission Enhancement Through a Curved Skull Model by MRFM

Considering the curved shape and non‐uniform thickness of the skull, we further model a realistic curved skull geometry based on Computed Tomography (CT) imaging to explore ultrasound transmission in the time domain where the acoustic attenuation coefficient is ignored. Due to the high impedance mismatch, the sound intensity decreasing to 46.5% when the ultrasonic wave at 0.309 MHz passing through the curved skull model (SC) only (**Figure** **3**a–c), while the sound intensity remains to 68.7% in the case of SC with the arrayed MRFM (Figure 3d–f). Due to the variations in the distance between the SC and each micropillar in the MRFM, resulting in inconsistent structural parameters across units and a weakened Fano resonance. Consequently, the enhanced transmittance effect is not prominent.

However, the flexibility of the MRFM allows it to conform precisely to the curved shape of the skull. Considering the conformal deformation of the MRFM upon attachment to the SC, the positions of micropillars within the MRFM would change adaptively. We carefully constrained the arrangement of micropillars according to the shape of SC and the fixed parameters of MRFM, as shown in Figure S7. Simulation results reveal a significant ultrasonic enhanced transmittance effect under this condition, as shown in Figure 3g–i. The results demonstrate that the sound intensity transmittance increases to 89.8%, showing an improvement of 43.4% compared to the case of SC only, while the amplitude of the sound pressure signal increases from 51.4% to 94.8% over time and then stabilizes, reflecting the steady evolution of Fano resonance. This strong Fano resonance effect arises from the consistent structural alignment between the MRFM and SC units, leading to enhanced ultrasonic transmission. Additionally, we calculate the sound intensity distribution for focused beam passing through SC, SC with arrayed MRFM, and SC with fitted MRFM, which align closely with the plane wave results, as shown in Figure S8. These findings confirm that strong Fano resonance and high transmission peaks are only achieved when MRFM conforms to the curved shape of the skull, highlighting the need for a flexible, water‐like substrate for MRFM.

Furthermore, the effect of individual micropillars’ displacement on the transcranial transmission was also conducted in Figure S9, showing that slight displacements (the displacement of the micropillars variation is smaller than that of 0.1 times the pitch of the micropillars) do not significantly affect the performance of the MRFM. However, when the displacement reaches 0.5 times the pitch of the micropillars, the highest transmittance decreases from 88% to 66%, establishing the periodic arrangement of micropillars as a critical fabrication parameter in MRFM's preparation.

To further investigate the ultrasound transmittance enhancement, we also simulated the conditions of the MRFM and the skull placed in the near‐field region of the transducer. As shown in the Figure S10, the simulation was conducted using a transducer with a diameter of 25 mm, operating at a frequency of 309 kHz. The results revealed that the acoustic pressure amplitude along the z‐axis is consistent with the radiation pattern of a typical piston source, where the near‐field region exhibits highly irregular and complex acoustic pressure distribution, and the far‐field region demonstrates progressively stable patterns. And the critical distance separating near‐field and far‐field regions z_g_ is 40 mm. Based on the fundamental model, the MRFM and skull were placed in direct contact with the transducer to assess the acoustic pressure distribution along the z‐axis. And the results revealed that the MRFM significantly enhances transcranial transmission within the range of z = 10–60 mm, corresponding to a depth of 1–50 mm beyond the skull. These results demonstrate that the MRFM can effectively improve transmittance even under near‐field conditions, further supporting its potential for practical applications in transcranial ultrasound.

### 2.4 Ultrasound Transmission Enhancement Through a Plate Skull Model with MRFM in Experimental Validation

**Figure 4:**
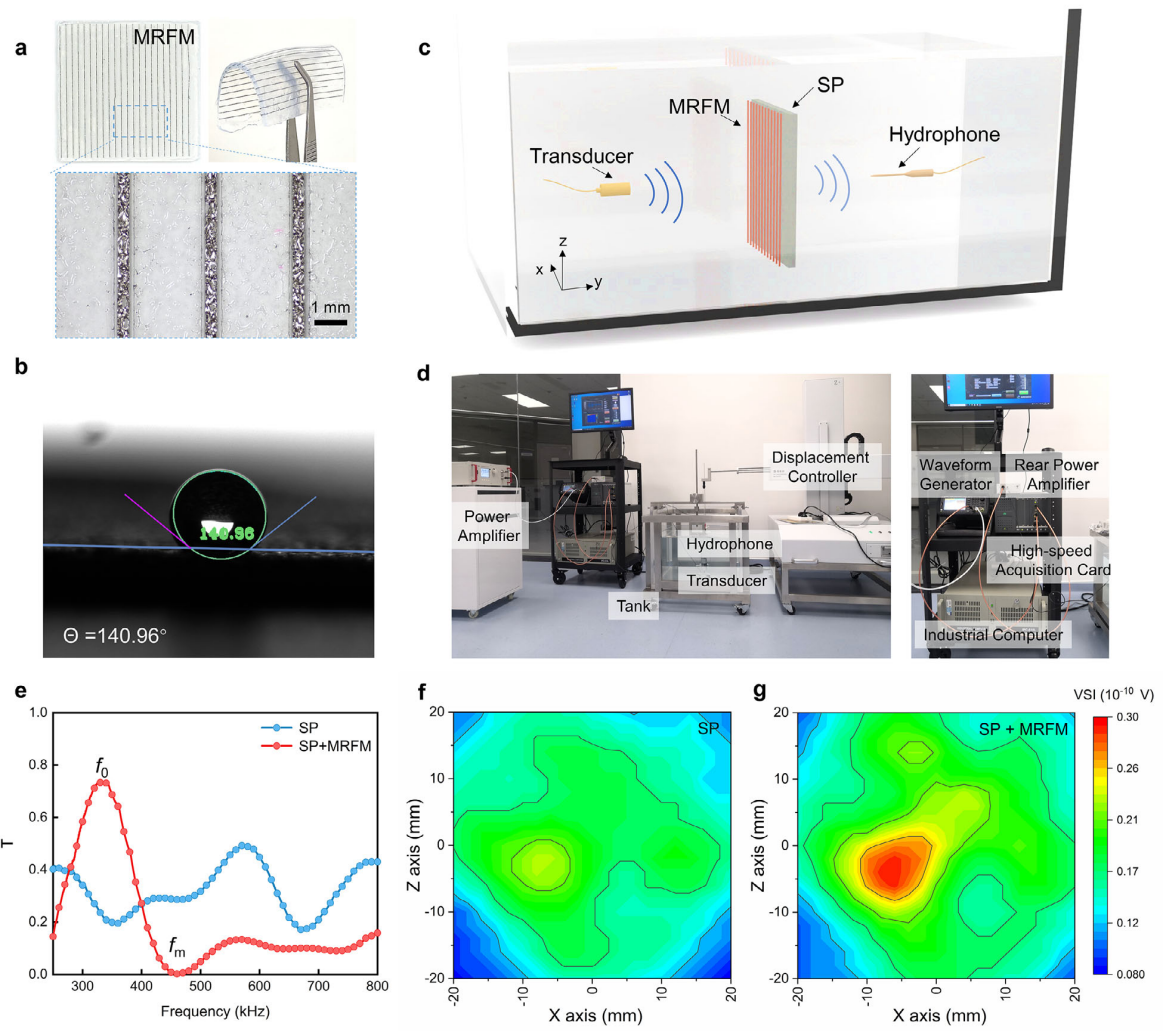
Ultrasound transmission experiments through a plate skull model (SP) with MRFM in water. a) The photograph and microscope image of prepared MRFM. b) The contact angle test for Nickel foam after hydrophobic treatment. c) The schematic of the experimental setup. d) The photograph of the ultrasound scanning system. Refer to the Methods section for detailed test steps. e) The transmission spectrum of ultrasonic wave passing through SP with and without the MRFM. f,g) The ultrasound scanning images. The cases of SP only (left), and MRFM installed in front of the SP (right) are shown. The voltage squared integral (VSI) is transferred from the voltage amplitude of the signal.

Based on the simulation results, we identify two key characteristics that the MRFM must possess periodically arranged low‐speed micropillars and a high‐speed flexible substrate. For the low‐speed micropillars, we selected 200‐µm‐thick nickel foam with porous and hydrophobic properties as the base material, and fabricated periodically arranged micropillars using a dicing machine, with each micropillars unit having a width of 200 µm. **Figure** **4**a shows a photograph of the MRFM and a corresponding microscope image at a local magnification. The prepared micropillars feature square cross‐section, and we demonstrate that MRFM with square Mie scatters similarly achieves a significant enhancement in transcranial transmission, as shown in in Figure S11. For the flexible substrate, agarose hydrogel was selected, whose acoustic properties are similar to those of water, as shown in Figure S13a. Its excellent flexibility is evident from Figure 4a. Detailed preparation methods can be found in the Methods section. Notably, the contact angle of the nickel foam is 140.96°, as shown in Figure 4b, indicating its hydrophobic nature, which effectively prevents the agarose hydrogel solution from penetrating the porous structure of the nickel foam.

To experimentally verify that MRFM significantly enhances transcranial transmission based on the Fano resonance, we created a plate skull model (SP) using epoxy resin and alumina (acoustic parameters of SP are shown in Figure S14 and Table S1). The prepared MRFM was tightly attached to the SP by the custom‐designed holder, and both were immersed in a water tank for acoustic transmission spectrum and scanning tests, as shown in Figure 4c,d. Detailed testing procedures are provided in the Methods section. The sound intensity transmittance for SP fluctuates between 20% and 40%, reflecting the intrinsic transmission properties of SP and the diffraction effects during the actual tests. In contrast, the transmission spectrum of the SP with MRFM shows a characteristic asymmetric peak indicative of Fano resonance, with the sound intensity transmittance at *f*_m_ (0.46 MHz) reaching 3.71%, and at *f*_0_ (0.33 MHz) reaching 73.3%, as shown in Figure 4e. To validate the transcranial ultrasound transmission enhancement, field scanning was conducted, with Figure 4f,g demonstrating a comparative analysis of ultrasound imaging through the SP with and without the MRFM. The results clearly show that the transmitted energy through the SP with MRFM is significantly higher than that passing through the SP only, confirming the enhanced transcranial transmission capability of the MRFM. Figure S15 also presents the simulations and experimental analyses of a specifically designed MRFM for enhancing ultrasound transmission through another plate skull model with a thickness of 5.86 mm, sound velocity of 2482 m s^−1^, and density of 1305 kg m^−^^3^. The results experimentally demonstrate that MRFMs can be tailored to specific skull parameters to significantly enhance transmittance. Additionally, the MRFM with designed parameters similarly enhances the ultrasound transmittance passing through a 0.35‐mm‐thick steel, as shown in Figure S16, indicating its potential applications in underwater acoustics.

## 3 Conclusion

To address the challenge of low ultrasound transmission through the skulls, this work proposes the use of MRFM to enhance transcranial transmission. Through simulations and experimental verification, we demonstrate that the artificially designed metamaterial generates Mie resonance at a specific frequency, which couples with a continuous state of the skull to form Fano resonance, enabling effective transcranial transmission at the target frequency *f*_0_.

The MRFM exhibits a simple structural design, and its geometric parameters (e.g., radius) can be precisely tuned to optimize transmission enhancement for skulls with varying acoustic properties. Furthermore, the MRFM inherent flexibility enables it to conform seamlessly to curved surfaces, thereby addressing the limitations of conventional rigid metamaterials. However, we acknowledge several limitations that warrant further investigations. While we have performed simulations to account for the three‐layer, anisotropic structure of skulls, the issue of anisotropy with elastoplastic characteristics of the skulls has not yet been incorporated. Additionally, we are currently unable to fabricate MRFM capable of enhancing transcranial transmission above 0.35 MHz constrained by manufacturing tolerances.

In future studies, we will systematically investigate the enhanced transcranial ultrasound performance of MRFM on more complex skull phantoms with heterogeneous and anisotropy properties. Additionally, we aim to refine the Fano resonance control equations within this complex framework and refine fabrication methods to enable MRFM operation at frequencies exceeding 0.5 MHz. We believe this work offers an innovative solution for improving ultrasound transmittance through skulls or other complex obstacles, and it holds significant potential for applications in transcranial ultrasound stimulation and underwater acoustics.

## 4 Experimental Section

### Transmission Simulation

Finite element simulations were conducted to investigate the wave transmission through the skull model with and without the MRFM using Comsol Multiphysics 6.1 software. Based on the CT image, the contour of the skull was outlined and imported into the software at an appropriate scale. The parameters and layout of the MRFM were then designed according to the acoustic characteristics and surface of the skull.

1) Frequency‐domain simulation: Frequency‐domain simulations were conducted to characterize the transcranial transmission behavior under steady state. A planar acoustic wave source was implemented by defining the two opposing boundaries of the water domain as dual‐port boundary conditions, enabling the calculation of the transmission spectrum through frequency‐domain analysis. To include the skull's intrinsic acoustic attenuation, the attenuations coefficient—incorporating frequency‐dependent absorption and scattering effects—was integrated into the simulations according to the reference.^[^ ^30^ ^]^ The wavenumber of the skull was described as:
(1) $$k_{s}=2\pi f/c_{s}-\;i\alpha$$
  where *α* is the attenuation coefficient. The attenuation coefficient can be set as:
(2) $$\alpha =2\pi f\times \;\mathrm{loss}/c_{s}$$
  where loss is independent of frequency. And the value of loss was adjusted in the simulations to ensure it closely matched the experimental results.

2) Time‐domain simulation: The ultrasonic source was strategically positioned at the boundary of the water medium. A perfectly matched layer (PML) absorption boundary condition was implemented on the other boundaries to ensure numerical accuracy and prevent boundary reflections. A critical simplification was introduced in the skull domain by omitting attenuation coefficients, since the complex frequency‐dependent attenuation characteristics of biological tissues pose significant challenges for precise time‐domain implementation. Through time‐resolved simulations, the spatiotemporal evolution of acoustic intensity distributions was systematically quantified.

### Preparation of the MRFM

The agarose hydrogel solution (Shanghai Yuanye BioTechnology Co.,Ltd) was prepared with a mass fraction of 3%. Subsequently, nickel foam (Yiminglong Co.Ltd., 0.2 mm) slices were sectioned into array columns measuring 0.2×0.2×70 mm, with an interval of 3 mm, using a dicing machine (Bojie Core Semiconductor Co.Ltd, LX‐3252). The agarose hydrogel solution, maintained at 90 °C, was poured into the mold, and the array column was quickly covered. When the agarose hydrogel solidified, it was turned over and encapsulated with agarose hydrogel solution. The MRFM was obtained with nickel foam Mie micropillars and agarose hydrogel flexible matrix. Figure S12 shows the detailed schematic. Additionally, to prevent dehydration, the MRFM was supposed to be stored in water when not in use. The dehydration process was presented in Figure S13b. This practice ensures its stability and maintains its water‐like properties for experimental purposes.

### Preparation of the Plate Skull Models

Epoxy resin A‐glue and B‐glue were mixed in a 1:1 ratio, followed by the addition of nano alumina powder with a mass fraction of 40%. The mixture was stirred thoroughly and poured into a silicone mold measuring 80×80×8 mm. It was then left to cure for 24 h. After curing, the surface was polished using sandpaper to obtain the 6.9‐mm‐thick plate skull model.

### Acoustic Performance Test

Both the ultrasonic 3D scanning field test and the ultrasonic transmission spectrum test were conducted using commercial ultrasonic testing platforms. Custom‐designed holder was employed to securely hold the MRFM and SP in place, ensuring the tight fit throughout the experiments.

Ultrasonic 3D scanning test method: A waveform generator (KEYSIGHT, 33500B) was used to transmit a 15‐cycle sinusoidal pulsed signal, which was amplified by 30 dB through a power amplifier (Aigtek ATA‐8202), making the ultrasonic transducer (Guangdong Goworld Co.,Ltd, 0.5P20) emit an ultrasound sinusoidal pulse. After passing through the sample, the ultrasonic waves were collected by a hydrophone (Precision Acoustics, NH0500), converted into electrical signals, and output to a high‐speed acquisition card (PXIe‐5122). The software algorithm (Shenzhen Boray Co.Ltd, BRC Motion Software & BRC Analysis Software) converted the electrical signals into voltage square integrals (VSI), while the displacement controller (Shenzhen Boray Co.Ltd, BRC8090) controlled the hydrophone position to perform 3D underwater ultrasonic scanning.

Ultrasonic transmission spectrum test method: A signal generator emitted a modulated pulse 𝐼(𝑡), which passed through the power amplifier, transducer, sample, and hydrophone, eventually reaching a digital storage oscilloscope. The modulated pulse 𝑇𝑟(𝑡) transmitted through the sample was recorded, and Fourier transform was applied to obtain the transmission spectrum of the sample.

## Conflict of Interest

The authors declare no conflict of interest.

## Supporting information



Supporting Information

## Data Availability

The data that support the findings of this study are available from the corresponding author upon reasonable request.
