# Supplementary material for: Achieving High‐Performance Transcranial Ultrasound Transmission Through Mie and Fano Resonance in Flexible Metamaterials
Source: Adv Sci (Weinh). 2025 Mar 26;12(19):2500170. doi: 10.1002/advs.202500170 (PMC12097009; doi:10.1002/advs.202500170)
Supplement: Supplementary file 1 — Supporting Information [file ADVS-12-2500170-s001.pdf]

ADVANCED SCIENCE
Open Access

Supporting Information

for *Adv. Sci.*, DOI 10.1002/advs.202500170

Achieving High-Performance Transcranial Ultrasound Transmission Through Mie and Fano Resonance in Flexible Metamaterials

Jie Chen, Bing Liu, Genshen Peng, Linming Zhou, Chengwei Tan, Jiale Qin, Juan Li, Zijian Hong, Yongjun Wu, Minghui Lu, Feiyan Cai and Yuhui Huang**

Supporting Information

Achieving High-Performance Transcranial Ultrasound Transmission through Mie and Fano Resonance in Flexible Metamaterials

Jie Chen, Bing Liu, Genshen Peng, Linming Zhou, Chengwei Tan, Jiale Qin, Juan Li, Zijian Hong, Yongjun Wu, Minghui Lu, Feiyan Cai, Yuhui Huang**

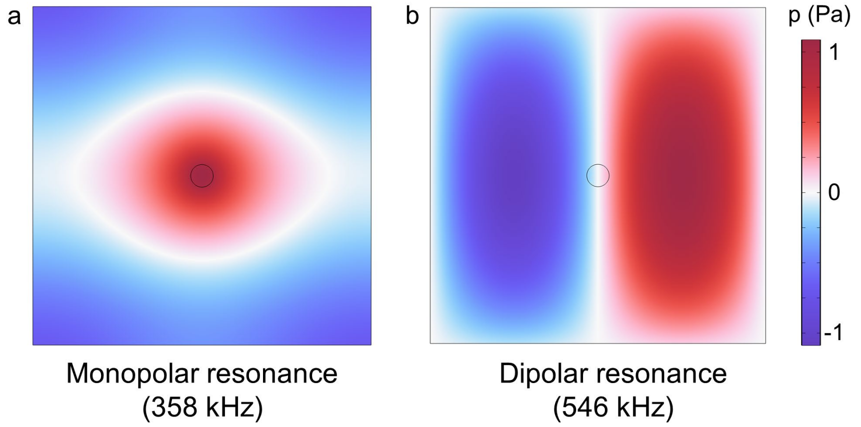

Figure S1. The eigenmodes of the MRFM. a) Monopolar resonance at 358 kHz and b) dipolar resonance mode at 546 kHz. In our work, the monopolar resonance contributes to the discrete state for Fano resonance.

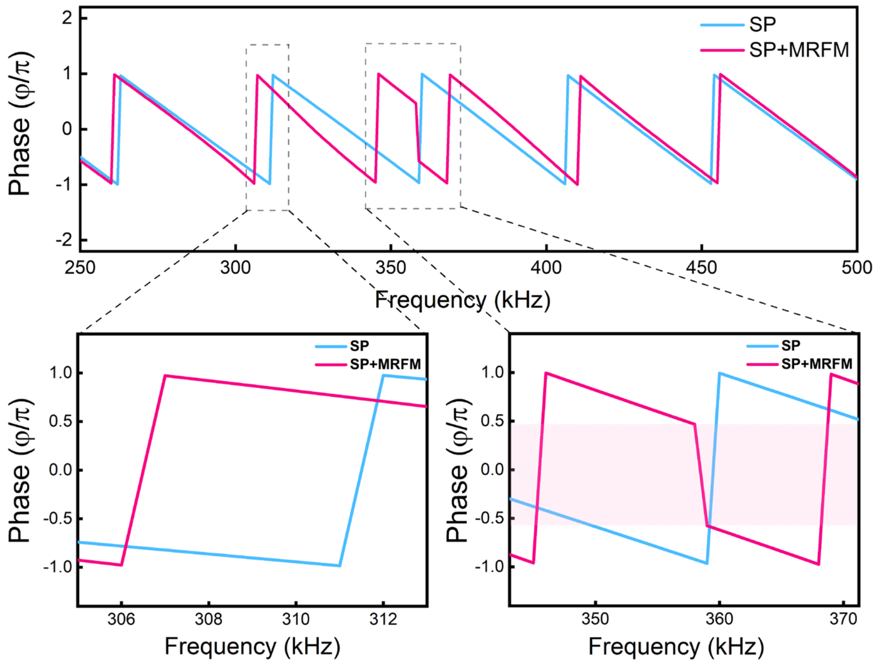

Figure S2. The simulation on the transcranial ultrasound transmission phase of SP model with and without the MRFM, which reveals that the phase undergoes a π-phase shift at the Mie resonance frequency f_m.

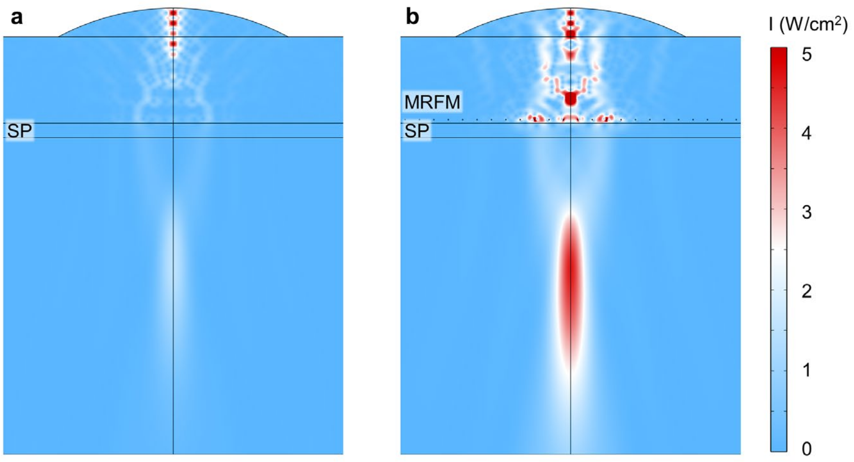

Figure S3. Ultrasound transmission simulations when focused beam passing through a plate skull model (SP) without and with the MRFM. a) The sound intensity distribution of the SP only. b) The sound intensity distribution of the SP with MRFM.

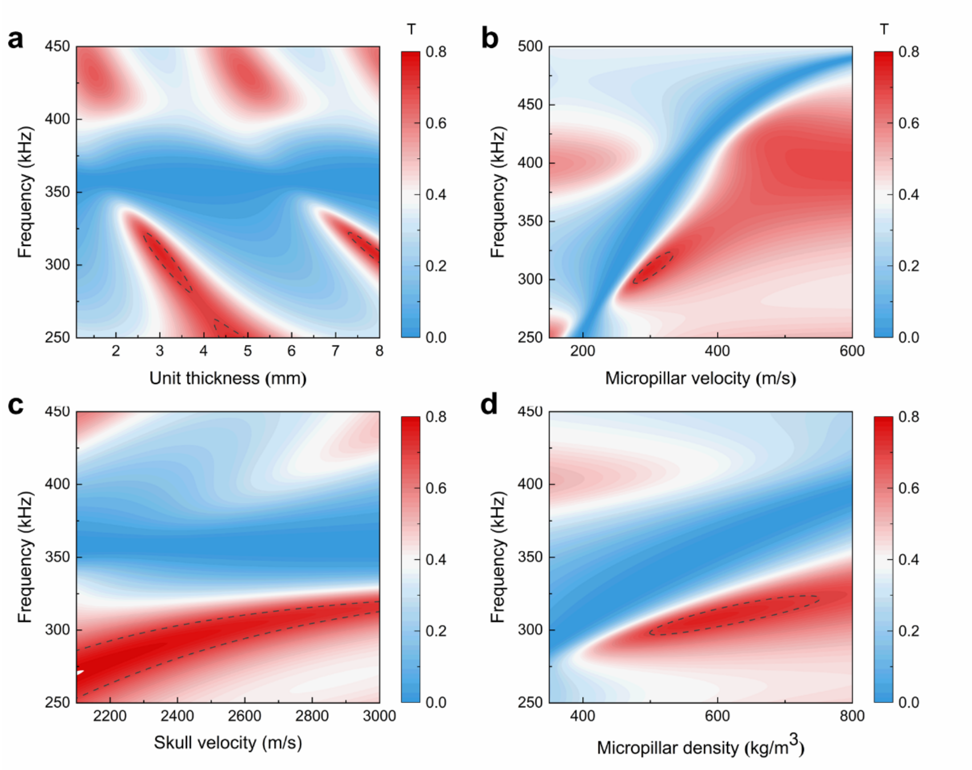

Figure S4. Ultrasound transmission simulations through the SP with MRFM of various parameters. a) The contour map depicting the influence of different unit thickness d_m, b) that of micropillar velocity, c) that of skull velocity, d) that of micropillar density. The area enclosed by the dotted line represents regions where the sound intensity transmittance exceeds 70%. The loss effect caused by the skull is included in the simulations. (Parameter control protocol: All non-target parameters are fixed at baseline values from Table S1)

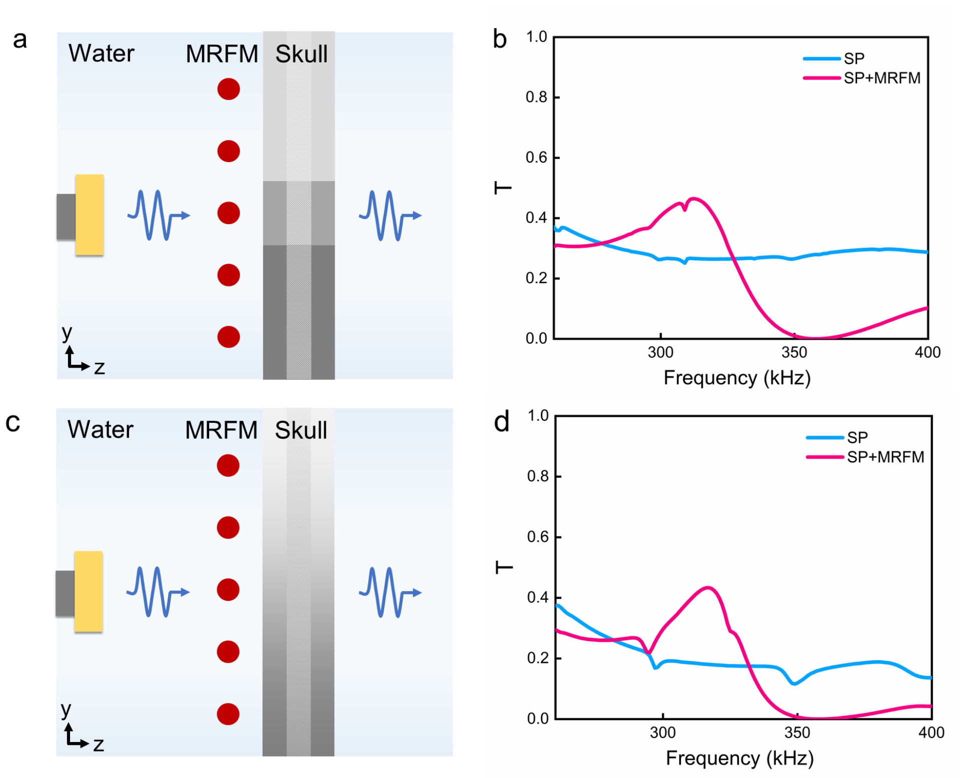

Figure S5. The simulation on the transcranial ultrasound transmittance of complex skull model with and without the MRFM. (a-b) Non-uniform density and sound speed properties in the three-layer skull model. (c-d) Gradient density and sound speed properties in the three-layer skull model.

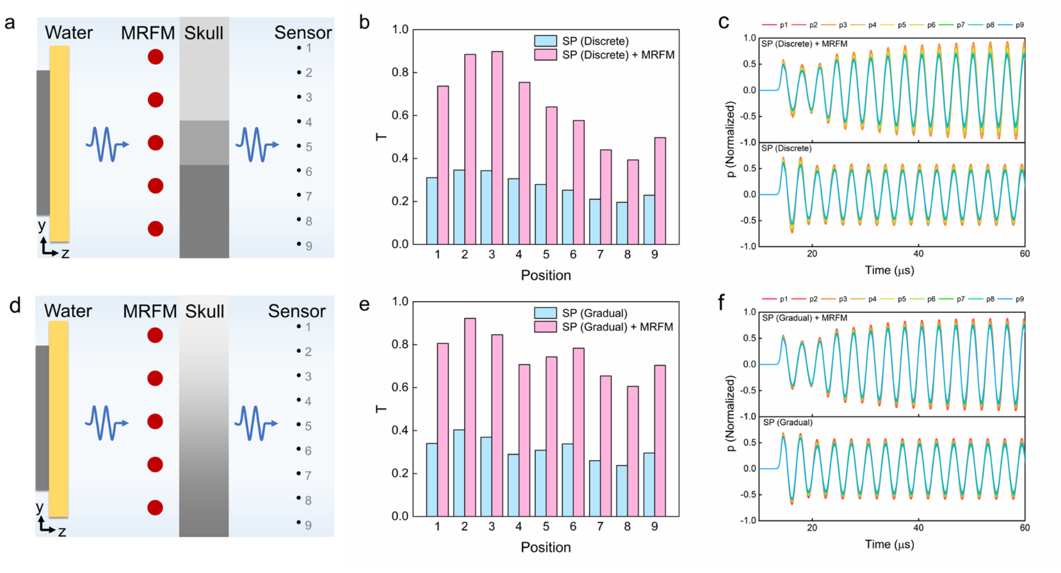

Figure S6. The simulation on the transcranial ultrasound transmittance of complex skull model with and without the MRFM. a-c) The transmission enhancement of the skull model with discrete density and sound speed properties at nine different areas. d-f) The transmission enhancement of the skull model with linearly gradual density and sound speed properties at nine different areas. The attenuation coefficients are ignored in the time-domain simulation conditions.

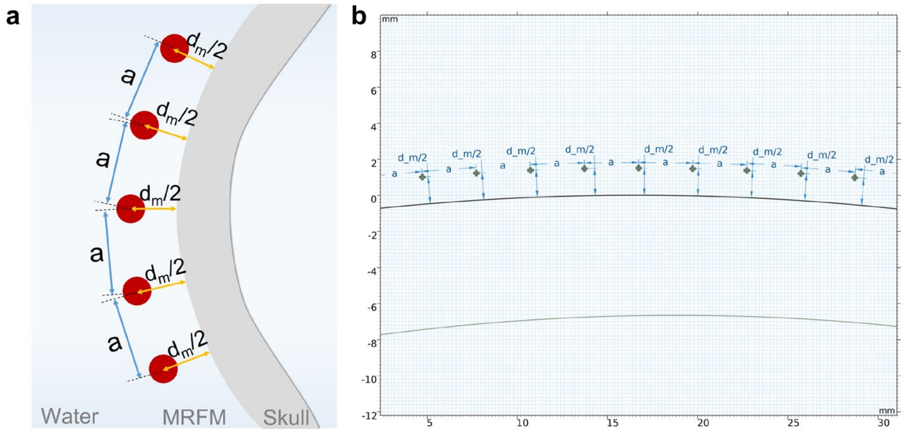

Figure S7. a) The schematic of the arrangements for the bend MRFM. b) Implementation of constraints on micropillar positions in simulations.

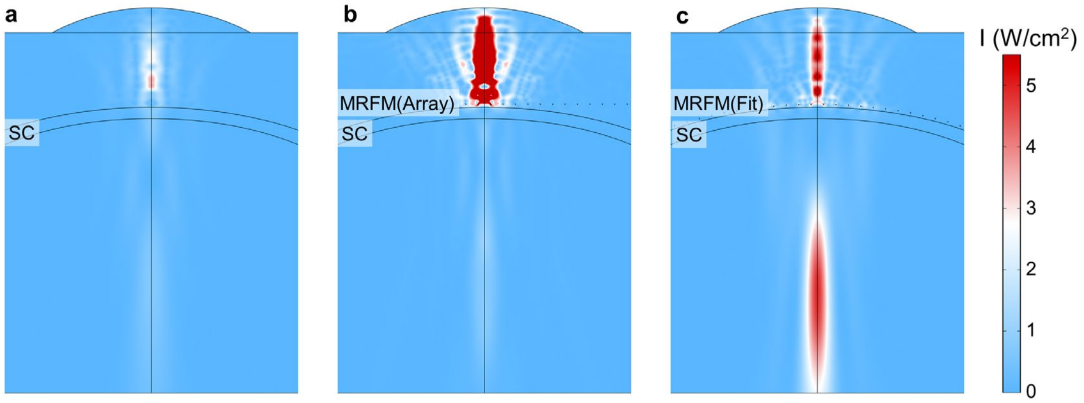

Figure S8. Ultrasound enhanced transmission simulations when focused beam passing through a curved skull model (SC) with MRFM. a-c) The simulated sound intensity distribution of focused ultrasound beam passing through SC only, SC with arrayed MRFM, and SC with fitted MRFM.

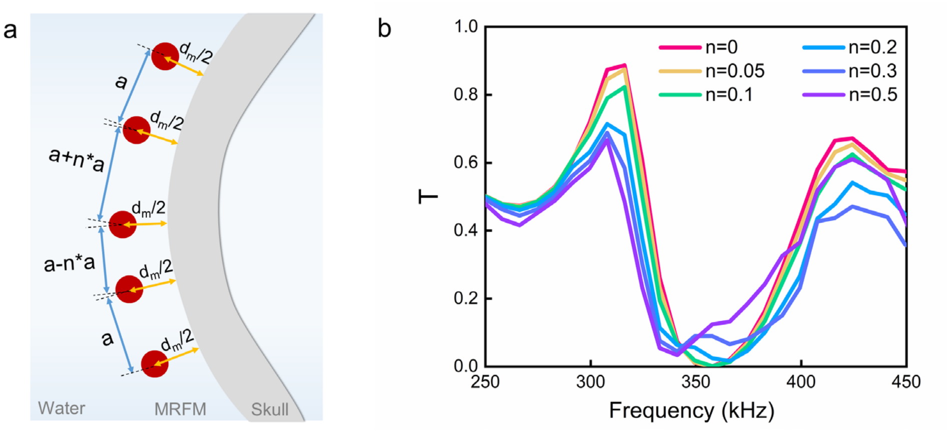

Figure S9. The effect of displacement of the micropillars on the ultrasound transmittance.

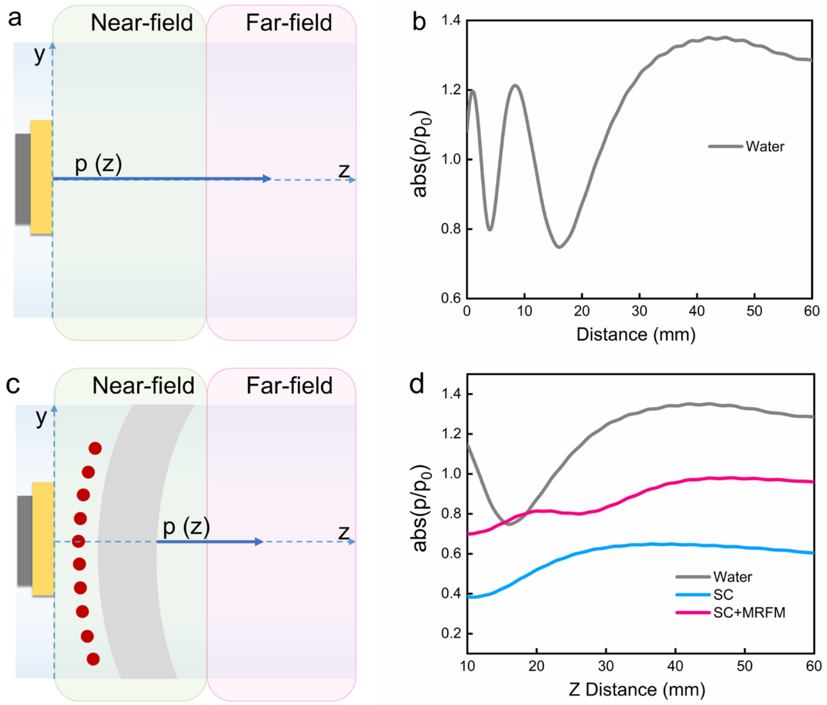

Figure S10. Transcranial transmission enhancement with MRFM when MRFM and the curved skull was placed in the near-field. a-b) Simulation on the near-field and far-field sound pressure distribution of a transducer. Here, p_0 represents a specific reference sound pressure, which is calculated based on the sound speed, density, and particle velocity at the reference point (0-point position). c-d) The sound pressure amplitude distribution along the z-axis when MRFM and the curved skull was placed in the near-field. The attenuation coefficients are included in the frequency-domain simulation conditions.

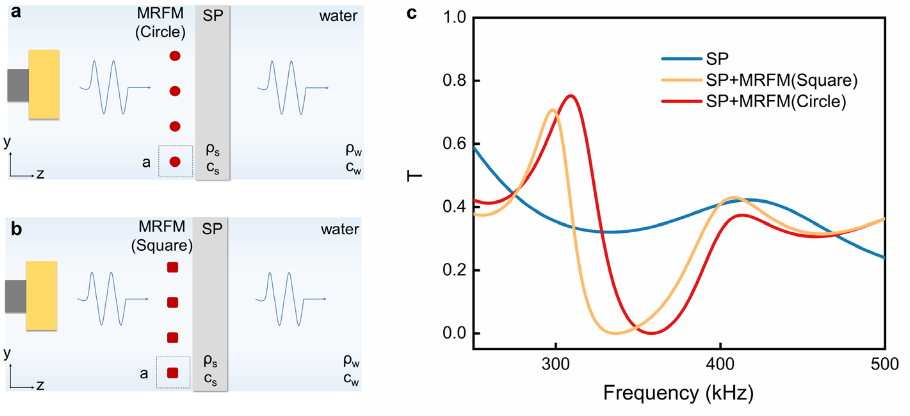

Figure S11. Transmission spectrum simulations of the SP with MRFM in different shapes. a) The schematic of ultrasound passing through the SP with MRFM, where the low-speed Mie scatters are in circle shape, and b) where the low-speed Mie scatters are in square shape. c) The simulated transmission spectrum of ultrasound passing through SP and MRFM with the different shape. The loss effect caused by the porous structure of the skull is included. The results demonstrate that MRFM with square Mie scatters also achieves a significant enhancement in transcranial transmission, offering valuable insights and guidance for future experiments.

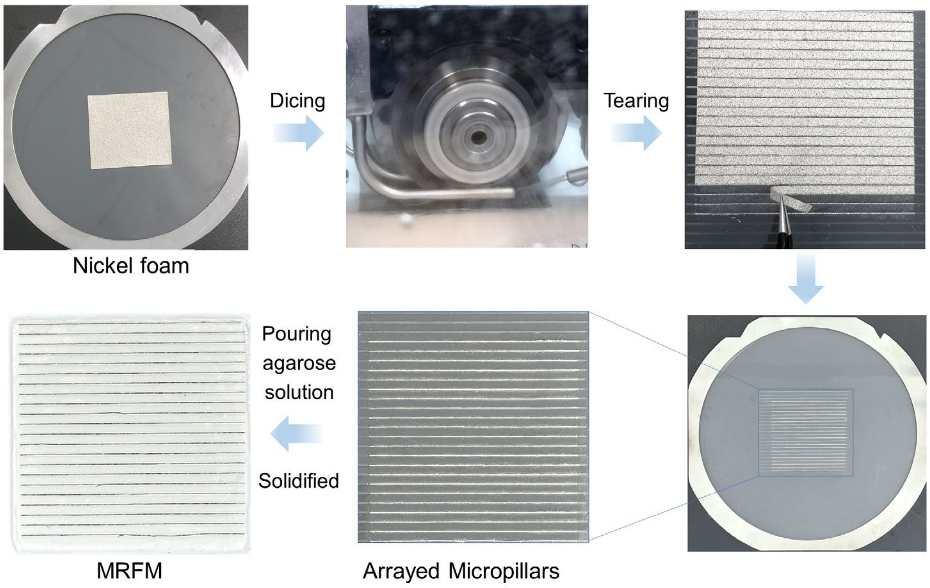

Figure S12. The preparation of MRFM. Refer to the Methods section for detailed preparation steps.

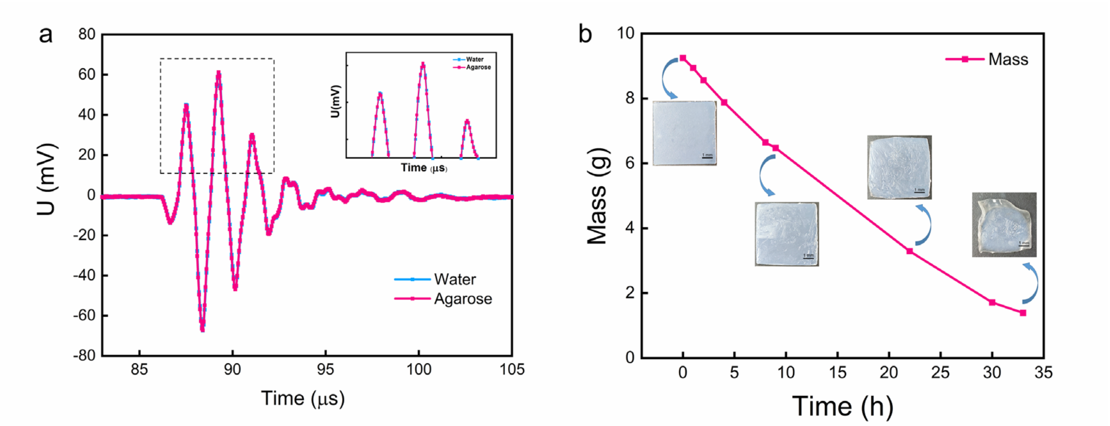

Figure S13. a) The transmittance and the sound velocity test of the water-like agarose hydrogel. b) The dehydration tests of the agarose hydrogel.

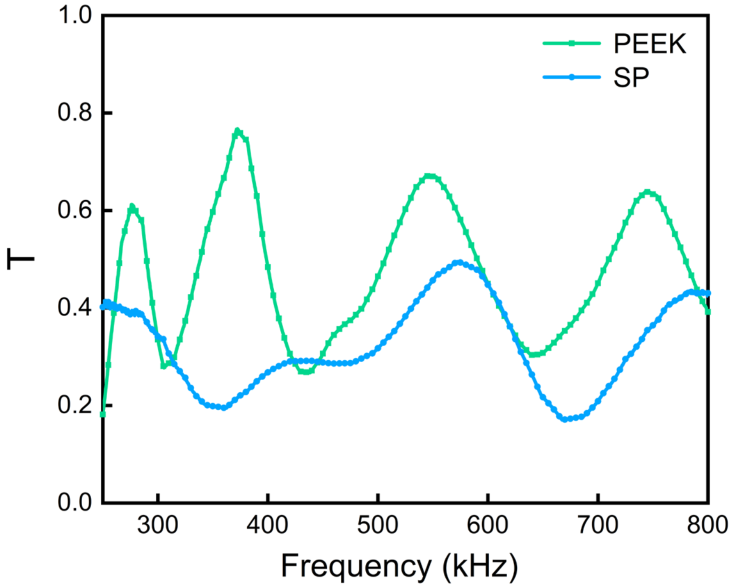

Figure S14. The ultrasound transmittance at 250-800 kHz for PEEK and SP (used in the experiments).

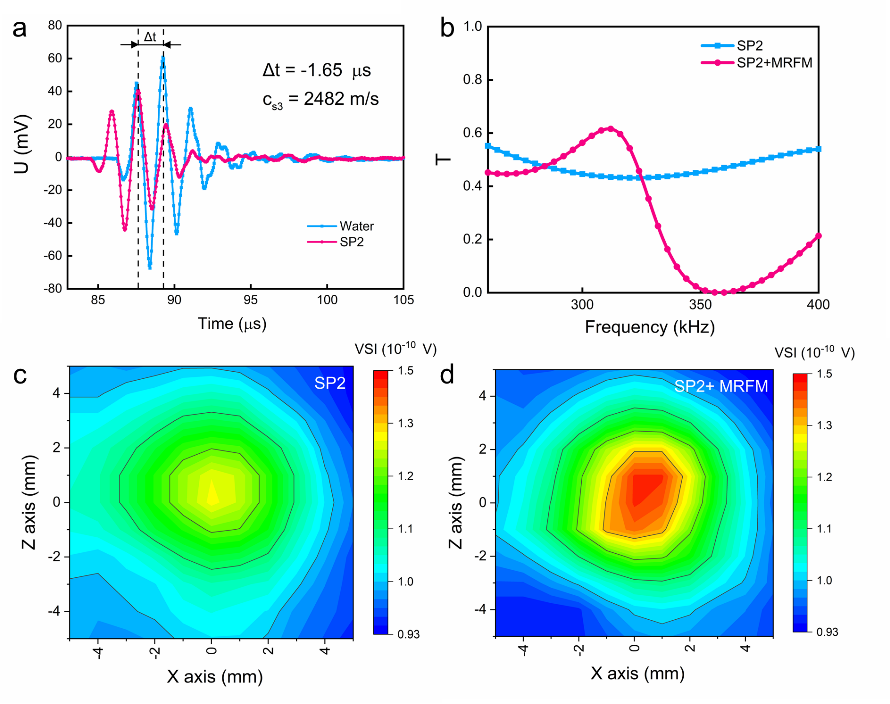

Figure S15. a) The sound velocity test result of the SP2. b) Results on the transmission spectrum for SP2 with and without the MRFM in simulation. c) The ultrasound scanning images of SP2 only, d) MRFM installed in front of the SP2. The voltage squared integral (VSI) is transferred from the voltage amplitude of the signal.

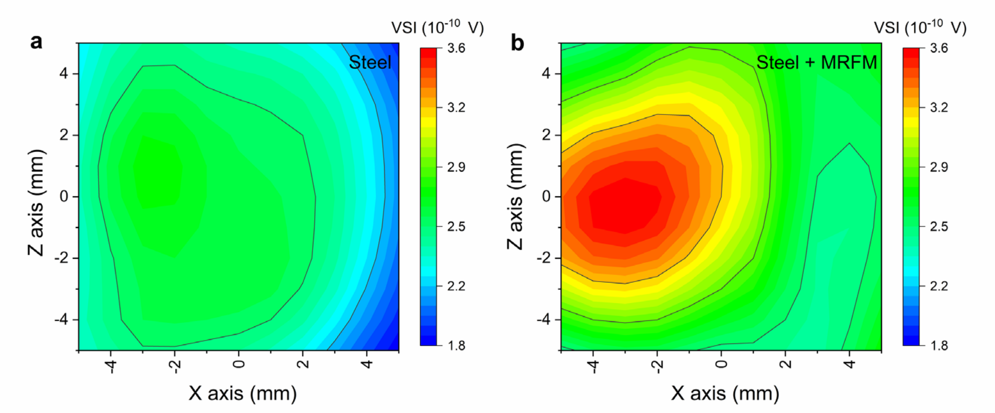

Figure S16. Experiments on enhanced ultrasound transmission passing through a 0.35-mm-thick steel plate with MRFM. a) The ultrasound scan images of a 0.35-mm-thick steel plate only. b) The ultrasound scan images of the MRFM installed in front of the steel plate. The voltage squared integral (VSI) is transferred from the voltage amplitude of the time signal. It demonstrates that MRFM can enhance the transmission of ultrasound passing through the steel plate, indicating its potential applications in underwater acoustics.

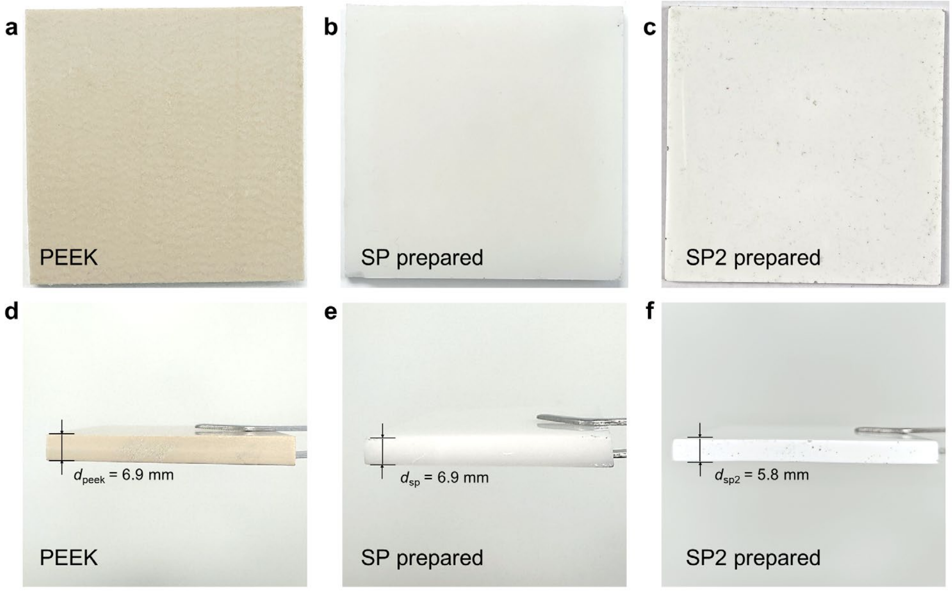

Figure S17. The photographs of PEEK and the plate skull model (SP) prepared in this work. Refer to the Methods section for detailed preparation steps.

Table S1. The structure and material parameters in simulations

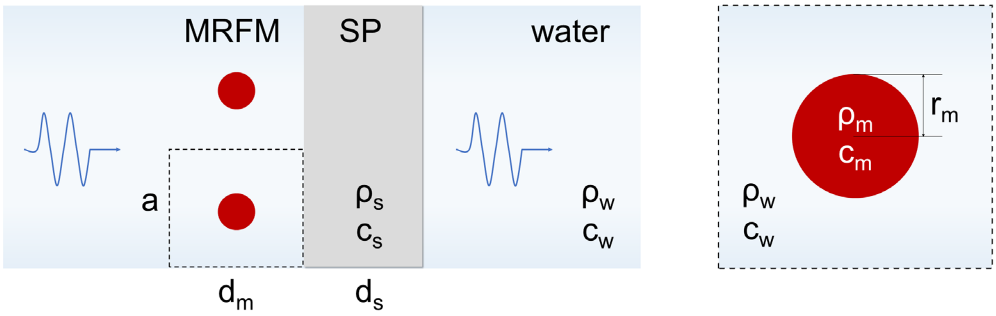

Name	Value	Description
ρ_w	1000 kg/m^3	The density of water
c_w	1500 m/s	The velocity of water
ρ_s	1370 kg/m^3	The density of SP
c_s	2751 m/s	The velocity of SP
d_s	6.9 mm	The thickness of SP
ρ_m	600 kg/m^3	The density of micropillars
c_m	300 m/s	The velocity of micropillars
r_m	100 μm	The radius of micropillars
d_m	3 mm	The thickness of MRFM
a	3 mm	The unit length of MRFM

Table S2. The comparison of MRFM's enhanced transmission performance through barriers with other methods

Study	Type of barriers	Strategy	Materials	Working Frequency	Outcomes
**Shen 2014** [1]	Aberrating layer (2000 kg/m^3, 2500 m/s)	Transformation Acoustic	Complementary metamaterials (unit cells formed by membranes and side branches with open ends)	50 kHz	Acoustic intensity increases from 28% to 88% (Sim.)
**Wang 2021** [2]	Low-impedance barrier	Transformation Acoustic	Complementary metamaterials (unit cells containing Helmholtz resonators and membranes)	276 kHz	Transmission intensity increases from 64% to 85% (Sim.)
**Li 2022** [3]	A 3D-printed skull model (1380 kg/m^3, 1615 m/s)	Transformation Acoustic	Phase-modulated complementary metamaterials (unit cells containing Helmholtz resonators)	113 kHz	Transmission enhancement of 24.8% (Sim.) and 22.4% (Exp.)
**Park 2023** [4]	1-mm-thick steel barrier	FP Resonance	Fabry-Perot resonance-tailoring panel (Rigid)	500 kHz	$SSIM_{No\ barrier}$ =0.6148, $SSIM_{Barrier}$ =0.0237, $SSIM_{Barrier + RTP}$ =0.5708 (Exp.)
**This work**	Skull phantom (1370 kg/m^3, 2751 m/s)	Fano Resonance	Mie-resonance flexible metamaterial consists of low-speed micropillars and flexible high-speed hydrogel	310 kHz	Acoustic intensity increases from 33.7% to 75.2% (Sim.) and from 20.6% to 73.3% (Exp.)

* "**Sim.**" represents the simulation results and "**Exp.**" represents the experimental results.

* "**SSIM**" means structural similarity index measure [5], (0 ≤SSIM≤ 1, 0: no similarity, 1: full similarity).

Note S1

The selection and preparation of the skull models

To experimentally verify that MRFM, in combination with the Fano resonance generated with the skull, significantly enhances transcranial transmission, we created a plate skull model ERP. Though PEEK was normally used as skull models in research, we would like to clarify that ERP was selected because its acoustic properties are closer to those of real skulls compared to PEEK, and it offers unique tunability that makes it more suitable for our study.

The transmittance of real skull is approximately reaching from 10% to 20%, while PEEK exhibits a transmittance reaching from 30% to 70%, which is significantly higher and less representative of skulls. In contrast, the ERP material we prepared has a transmittance reaching from 20% to 40%, as shown in Figure S14, which is much closer to that of real skulls. This low transmittance provides a more realistic baseline for our study, allowing us to explore the potential of ultrasonic metamaterials to enhance transmittance in a skull-like environment.

Additionally, ERP is a composite material composed of epoxy resin and alumina powder, which allows us to precisely adjust its acoustic properties by varying the mass fraction of alumina powder. This tunability enables us to control key parameters such as density, sound velocity, and acoustic impedance, making ERP a versatile and adaptable skull phantom material.

By using ERP, we aim to mimic the low ultrasonic transmittance of real skulls more accurately. The photographs of PEEK and the plate skull model (SP) prepared in this work are shown in Figure S17.

References

[1] C. Shen, J. Xu, N. X. Fang, Y. Jing, Anisotropic complementary acoustic metamaterial for canceling out aberrating layers. *Phys. Rev. X* 2014, **4**, 04103.

[2] J.Y. Wang, F. Allein, N. Boechler, J. Friend, O. Vazquez-Mena, Design and fabrication of negative-refractive-index metamaterial unit cells for near-megahertz enhanced acoustic transmission in biomedical ultrasound applications. *Phys. Rev. Appl.* 2021, **15**, 024025.

[3] L. C. Li, Y. F. Diao, H. J. Wu, W. K. Jiang, Complementary acoustic metamaterial for penetrating aberration layers. *ACS APPL MATER INTER* 2022, **14**, 28604-28614.

[4] C. I. Park, S. Choe, W. Lee, W. Choi, M. Kim, H. M. Seung, Y. Y. Kim, Ultrasonic barrier-through imaging by Fabry-Perot resonance-tailoring panel. *Nat Commun* 2023, **14**, 7818.

[5] A. Horé and D. Ziou, "Image Quality Metrics: PSNR vs. SSIM," 2010 20th International Conference on Pattern Recognition, Istanbul, Turkey, 2010, pp. 2366-2369, doi: 10.1109/ICPR.2010.579.
